# Outcomes of myocarditis in systemic sclerosis: A 3-year follow-up

**DOI:** 10.1515/rir-2024-0015

**Published:** 2024-07-15

**Authors:** Ajanee Mahakkanukrauh, Chingching Foocharoen, Narumol Chaosuwannakit, Siraphop Suwannaroj, Patnarin Pongkulkiat, Tippawan Onchan, Burabha Pussadhamma

**Affiliations:** Rheumatology Unit, Department of Medicine, Faculty of Medicine, Khon Kaen University, Khon Kaen, Thailand; Department of Radiology, Faculty of Medicine, Khon Kaen University, Khon Kaen, Thailand; Cardiology Unit, Department of Medicine, Faculty of Medicine, Khon Kaen University, Khon Kaen, Thailand

**Keywords:** systemic sclerosis, scleroderma and related disorders, myocarditis, myocardium inflammation, cardiac magnetic resonance imaging

## Abstract

**Background and Objectives:**

The clinical course, the outcomes of myocarditis, and the imaging progression of cardiac magnetic resonance imaging (MRI) in systemic sclerosis (SSc) are still unknown. We aimed at defining changes in cardiac MRI findings, the clinical course, and the outcomes of SSc patients previously defined as having myocarditis by cardiac MRI. Methods: This prospective cohort study included SSc patients, who had previously been diagnosed with myocarditis through cardiac MRI at the Scleroderma Clinic of Khon Kaen University, between 2018 and 2020 and had had annual follow-ups of cardiac MRI for at least 3 years. Data on demographics, clinical characteristics, cardiac MRI findings, treatment regimens, and outcomes were collected. Serial cardiac MRI on a yearly basis was analyzed to assess changes in myocardial involvement over the 3-year period.

**Results:**

Ten SSc patients diagnosed with myocarditis via cardiac MRI were included. Most belonged to the diffuse cutaneous subset with a mean age of 58.3±8.6 years and were mildly symptomatic. Initial cardiac MRI findings showed myocardial edema and hyperemia in all patients and eight patients had had pre-existing myocardial scars, suggesting disease chronicity. Treatment for concomitant interstitial lung disease involved steroids with either cyclophosphamide or mycophenolate mofetil in 6 patients. Outcomes of myocarditis were stable, improving, and worsening in 4, 4, and 2 patients, respectively. There was no complete resolution of the cardiac MRI indices for myocarditis, and none had had major cardiac events.

**Conclusion:**

Although SSc myocarditis on cardiac MRI may improve or show stability, the changes remained persistent. Among patients with SSc and mildly symptomatic myocarditis, the efficacy of steroids and immunosuppressive therapy is inconclusive. Over a 3-year follow-up, the prognosis had been acceptably good with no cardiac events.

## Introduction

Systemic sclerosis (SSc) is a rare autoimmune disease characterized by extensive fibrosis and vasculopathy affecting various organs throughout the body, leading to organ dysfunction.^[1]^ Myocarditis in SSc patients represents a serious manifestation that can lead to fatal outcomes.^[2]^ The clinical presentation of cardiac involvement in SSc varies widely, ranging from asymptomatic cases to palpitations, with approximately one-fourth of patients presenting with heart failure.^[3]^ Early detection of myocarditis is crucial since it offers an opportunity for timely intervention to prevent myocardial fibrosis.^[4]^ However, effective treatment strategies for myocarditis in SSc patients remain elusive.

The clinical course, cardiac magnetic resonance imaging (MRI) findings over time, and the long-term outcomes of myocarditis in SSc patients have not been thoroughly elucidated. Previous studies on cardiac complications and outcomes have primarily focused on myocardial perfusion defects and cardiac inflammation for diagnostic purposes only. Therefore, our study aimed at evaluating changes in cardiac MRI findings, the clinical course, and the outcomes of SSc patients with confirmed myocarditis on cardiac MRI.

## Methodology

A prospective cohort study with retrospective analysis was conducted on SSc patients diagnosed with myocarditis by cardiac MRI between 2018 and 2020 at the Scleroderma Clinic of Srinagarind Hospital at Khon Kaen University, who had undergone annual follow-up cardiac MRI for at least 3 years. The demographic, clinical data, laboratory results, and echocardiogram findings at the baseline and annually during cardiac MRI were retrieved from the medical records.

The type of MRI scanner was a 1.5 Tesla magnetic resonance (MR) scanner (Siemens Medical Solution, Erlangen, Germany) and the cardiac MRI procedure was comprised of several steps. Initially, an electrocardiogram (ECG)-triggered dark-blood-prepared half-Fourier acquisition single-shot turbo spin-echo (HASTE) sequence was utilized, covering the entire heart in the axial orientation. Subsequently, four-chamber and two-chamber views, along with contiguous short-axis images of the entire heart, were obtained using a fast imaging steady-state free precession (trueFISP) cine sequence. Oblique orientation images were also captured to further investigate any suspicious areas.

Following the injection of 0.2 mmol/kg of gadolinium diethylene triamine pentaacetic acid (Magnevist; Schering, Berlin, Germany) at a flow rate of 2 mL/sec, breath-hold ECG-triggered 2D inversion recovery turbo FLASH images of four-and two-chamber views were acquired. This was followed by repeated three-dimensional (3D) inversion recovery turbo FLASH sequences in the short-axis orientation. Additional oblique slices were obtained in patients with suspicious findings using either 2D or 3D inversion recovery turbo FLASH sequences.

Image acquisition occurred immediately after the contrast material had been injected and up to 15 min afterward for myocardial delayed enhancement assessment. The 2D sequence acquired single-slice images with a slice thickness of 8 mm, while the 3D sequence was able to capture up to 24 slices with a 4 mm slice thickness in one breath-hold. This was achieved using a shorter time of repetition (TR), partial Fourier reconstruction, z-axis interpolation, and a longer data acquisition window to improve speed, with 77 k-space lines per heartbeat.

The total imaging time, including patient positioning, had ranged from 45 to 60 min.

The parameters of cardiac MRI (cMRI), including the degree of myocardial edema, hyperemia, fibrosis, and ventricular function at the 3-year mark, were compared with the baseline cMRI.

The outcomes of stable, improved, and worsening cMRI parameters were determined based on the assessment of experienced radiologists, who were blinded to the clinical status of the patients.

### Operational Definitions

The diagnosis of SSc was based on 2013 American College of Rheumatology/European League Against Rheumatism (ACR/EULAR) Classification Criteria for Systemic sclerosis,^[5]^ and SSc was classified as limited cutaneous (lcSSc) and diffuse cutaneous (dcSSc) by the extent of skin involvement according to LeRoy *et al*.^[6]^ Patients, who had shown skin involvement limited to areas below the elbows and knees, including the face and neck were classified as lcSSc, and those patients, who had had skin involvement which extend proximally beyond the elbows and knees, involving the trunk were classified as dcSSc. The patient’s functional class was determined using the New York Heart Association (NYHA) classification system, which categorizes them into one of four functional classes (FC). Elevated N-terminal pro-brain natriuretic peptide (NT-ProBNP) levels are categorized based on age-specific cutoff values. For those patients under 50 years old, the threshold is set at > 450 ng/L, for those aged 50–75 years, it is > 900 ng/L, and for patients over 75 years old, it is > 1800 ng/L.^[7]^ An elevation in high-sensitivity cardiac troponin-T (hs-cTnT) is defined as a level exceeding 14 ng/L. Elevated erythrocyte sedimentation rate (ESR) and C-reactive protein (CRP) levels were defined as follows: ESR > 25 mm/hr and CRP > 5.0 mg/L. Pulmonary arterial hypertension (PAH) was defined from right heart catheterization at rest as a mean pulmonary arterial pressure (mPAP) of > 20 mmHg, accompanied by a pulmonary arterial wedge pressure (PAWP) of ≤15 mmHg, and a pulmonary vascular resistance (PVR) of > 2 Wood units.^[8]^ Major cardiac events consisted of congestive heart failure, pulmonary hypertension, symptomatic cardiac arrhythmia, cardiac tamponade, and cardiac arrest.

Myocarditis diagnoses were defined by using the 2018 updated Lake Louise Cardiac MRI Diagnostic Criteria for Suspected Myocarditis^[9]^ if 2 of the 2 following criteria were presented:

1. T_2_-based imaging
  - Regional high T_2_ signal intensity or
  - Global T_2_ signal intensity ratio > 2.0 on T_2_ weighted CMR (cardiovascular magnetic resonance) imaging (myocardium versus skeletal muscle) or
  - Regional or global increase of myocardial T_2_ relaxation time.
2. T_1_-based imaging
  - Regional or global increase of native myocardial T_1_ relaxation times or
  - Extracellular volume (ECV) or
  - Areas with high signal intensity in a non-ischemic distribution pattern on late gadolinium enhancement (LGE) imaging

Supporting criteria

- Pericardial effusion or
- High pericardial signal intensity on T_2 or_ T_2_-mapping or LGE imaging, or
- Left ventricular wall motion abnormality on cine imaging

### Statistical Analysis

Descriptive demographic data analysis was performed using the appropriate descriptive statistics. Continuous data was presented as means with standard deviations, while the categorical data was presented as proportions or percentages. All data analyses were performed using STATA version 11.2 (StataCorp., TX, USA).

## Results

A total of 10 SSc patients previously defined as having myocarditis underwent and completed their annual 3-year followup cardiac MRI. The majority of patients were in the diffuse cutaneous subset (70%) and were female (60%). The mean age of the patients was 58.3±8.6 years. The clinical characteristics of the patients at the baseline are provided in Table 1.

**Table 1:** Baseline demographic data

Clinical characteristic	Number
Female, *n* (%)	6 (60)
Age (years), mean ± SD	58.3 ± 8.6
Diffuse cutaneous SSc subset, *n* (%)	7 (70)
Duration of disease at myocarditis diagnosis (years), mean ± SD	4.8 ± 1.1
Anti-topoisomerase I positive, *n* (%)	10 (100)
Anti-centromere positive, *n* (%)	1 (10)
SSc clinical characteristics	
Functional Class	
FC I, *n* (%)	1 (10)
FC II, *n* (%)	8 (80)
FC III, *n* (%)	1 (10)
Raynaud’s phenomenon, *n* (%)	2 (20)
Digital ulcer, *n* (%)	0 (0)
Digital gangrene, *n* (%)	0 (0)
Telangiectasia, *n* (%)	7 (70)
Calcinosis cutis, *n* (%)	1 (10)
Salt and pepper appearance, *n* (%)	6 (60)
Edematous skin, *n* (%)	0 (0)
Tendon friction rub, *n* (%)	0 (0)
Hand deformity, *n* (%)	6 (60)
Synovitis, *n* (%)	0 (0)
Esophageal involvement, *n* (%)	3 (30)
Stomach involvement, *n* (%)	3 (30)
Intestinal involvement, *n* (%)	2 (20)
Interstitial lung disease	6 (60)
Renal crisis	0 (0)
Pulmonary hypertension	1 (10)
Echocardiogram	
LVEF < 45%	0 (0)
TRVmax > 2.8 m/s	1 (10)
Pericardial effusion	8 (80)
Laboratory	
High NT-proBNP	0 (0)
High ESR	7 (70)
High CRP	3 (30)
High hs-cTnT	7 (70)

CRP, C-reactive protein; ESR, erythrocyte sedimentation rate; FC, Functional Class; hs-cTnT, high-sensitivity cardiac troponin-T; LVEF, left ventricular ejection fraction; NT-proBNP, N-terminal pro-brain natriuretic peptide; SD, standard deviation; SSc, systemic sclerosis; TRVmax, tricuspid regurgitation maximum velocity.

During follow-up, one patient and five patients received immunosuppressants for interstitial lung disease (ILD) treatment at 8 months and 3 years after the first cardiac MRI, respectively, whereas four patients did not need to receive such therapy. At the 3-year follow-up, three patients had shown an improved NYHA FC, while 7 patients had had a stable NYHA FC, no patients had shown an left ventricular ejection fraction (LVEF) < 45%, and the hs-cTnT levels had seemingly improved or had been stable in 8 patients. There was one incidence of pulmonary hypertension-associated interstitial lung disease (PH-ILD) that had occurred in one patient 3.5 years after the first cardiac MRI in which mycophenolate mofetil was administered for treatment of ILD shortly before establishing diagnosis of pulmonary hypertension. There was also one patient, who had developed progressive ILD, although cyclophosphamide and mycophenolate mofetil had been used for treatment. However, none of the major cardiac events had occurred in any of the patients. Clinical courses, cardiac MRI findings, and cardiac outcomes of patients during follow-up are shown in Table 2.

**Table 2:** Clinical course, cardiac MRI findings, and cardiac outcome at diagnosis and at 3-year follow-up

Case	At diagnosis						Immunosuppressant	At 3-year follow-up						Cardiac outcomes
	
	FC	LVEF (%)	Hs-cTnT (ng/L)	Cardiac MRI finding				FC	LVEF (%)	Hs-cTnT (ng/L)	Cardiac MRI finding			
	
				Edema	Hyperemia	Scar					Edema	Hyperemia	Scar	
1	II	76.9	16.3	Present	Present	Absent	Cyclophosphamide for ILD treatment (starting at 3 years after 1^st^ cardiac MRI)	II	67.0	3.5	Present/ same	Present/same	Absent/same	None
2	III	71.1	95.4	Present	Present	Present	None	I	56.0	21.5	Present/ same	Present/same	Present/ same	None
3	II	63.9	396.00	Present	Present	Present	Cyclophosphamide for ILD treatment (starting at 3 years after 1^st^ cardiac MRI)	I	73.3	13.9	Present/ Improving	Present/ Improving	Present/ same	None
4	II	64.9	3.0	Present	Present	Present	Mycophenolate for ILD treatment (starting at 3 years after 1^st^ cardiac MRI)	II	78.8	19	Present/ Worsening	Present/ Worsening	Present/ same	None
5	II	70.9	49.1	Present	Present	Absent	Mycophenolate for ILD treatment (starting at 3 years after 1^st^ cardiac MRI)	II	50.0	38.3	Present/ Worsening	Present/ Worsening	Present/ Worsening	PH-ILD^*^ 3.5 years after 1st cardiac MRI
6	II	75.2	29.7	Present	Present	Present	Cyclophosphamide for ILD treatment (starting at 3 years after 1^st^ cardiac MRI) for 1 year, then mycophenolate	I	69.8	27.3	Present/ Improving	Present/ Improving	Present/ same	None but progressive ILD
7	I	78.4	3.0	Present	Present	Present	None	I	77.4	<3.0	Present/ Improving	Present/ Improving	Present/ same	None
8	II	65.4	66.0	Present	Present	Present	None	II	58.8	8.6	Present/ Improving	Present/ Improving	Present/ same	None
9	II	68.2	98.5	Present	Present	Present	None	II	73.7	40.7	Present/ same	Present/same	Present/ same	None
10.	II	72.5	9.5	Present	Present	Present	Cyclophosphamide for ILD treatment (starting at 8 months after 1^st^ cardiac MRI) for 1.5 year, then mycophenolate	II	70.1	59.0	Present/ same	Present/same	Present/ same	None

^*^Right heart catheterization revealed mean pulmonary arterial pressure 25 mmHg, pulmonary arterial wedge pressure 9 mmHg, and pulmonary vascular resistance 2.8 Wood Units, and cardiac output 5.59 L/min. FC, Functional Class; hs-cTnT, high-sensitivity cardiac troponin-T; ILD, interstitial lung disease; LVEF, left ventricular ejection fraction; MRI, magnetic resonance imaging; PH, pulmonary hypertension.

According to the status of myocarditis (classified by the cardiac MRI results) at the 3-year follow-up, stable myocarditis, improving myocarditis, and worsening myocarditis were found in 4, 4, and 2 patients, respectively. Treatment with immunosuppressants (primarily aiming for ILD treatment) was equally administered in 2 patients among each group of myocarditis status. There was no obvious trend of changes in NYHA FC, LVEF, and hs-cTnT between the initial diagnosis and the 3-year follow-up in any group of myocarditis status (Table 3).

**Table 3:** Changes of FC, LVEF, and hs-cTnT according to myocarditis status classified by cardiac MRI at 3-year follow-up

Stable myocarditis							Improving myocarditis							Worsening myocarditis						
		
Case	FC		LVEF(%)		Hs-cTnT (ng/L)		Case	FC		LVEF (%)		Hs-cTnT (ng/L)		Case	FC		LVEF(%)		Hs-cTnT (ng/L)	
	At diagnosis	3-year followup	At diagnosis	3-year followup	At diagnosis	3-year followup		At diagnosis	3-year followup	At diagnosis	3-year followup	At diagnosis	3-year followup		At diagnosis	3-year followup	At diagnosis	3-year followup	At diagnosis	3-year followup
1	II	II	76.9	67.0	16.3	3.5	3	II	I	63.9	73.3	396.0	13.9	4	II	II	64.9	78.8	3.0	19
2	III	I	71.1	56.0	95.4	21.5	6	II	I	75.2	69.8	29.7	27.3	5	II	II	70.9	50.0	49.1	38.3
9	II	II	68.2	73.7	98.5	40.7	7	I	I	78.4	77.4	3.0	<3.0							
10	II	II	72.5	70.1	9.5	59.0	8	II	II	65.4	58.8	66.0	8.6							

FC, Functional Class; hs-cTnT, high-sensitivity cardiac troponin-T; LVEF, left ventricular ejection fraction.

**Figure 1:**
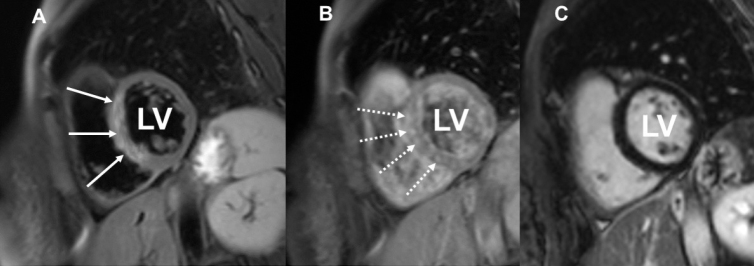
Cardiac MRI of a 38-year-old-woman with systemic sclerosis at baseline regional high T2 signal intensity at mid anteroseptal and inferoseptal segments of the left ventricle (A: white arrows), patchy mid wall non-ischemic pattern on early gadolinium enhancement sequence (B: dashed arrows), and no abnormal signal intensity of myocardium on late gadolinium enhancement (C). (LV: Left ventricle).

**Figure 2:**
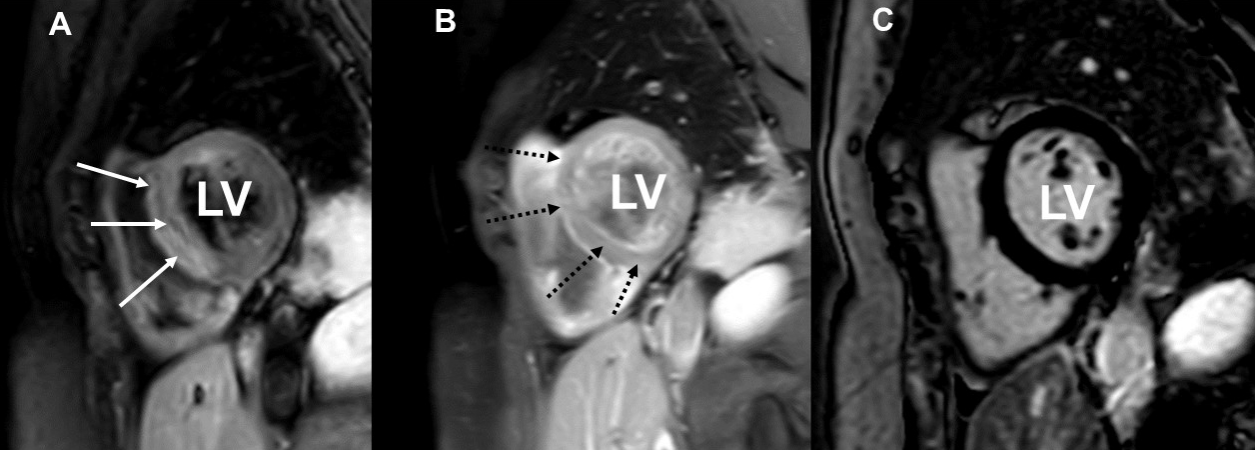
The three-year follow-up cardiac MRI of a 41-year-old-woman with systemic sclerosis demonstrated persistent regional high T2 signal intensity at mid anteroseptal and inferoseptal segments of the left ventricle (A: white arrows), patchy mid wall non-ischemic pattern on early gadolinium enhancement sequence (B: dashed arrows), no abnormal signal intensity of myocardium on late gadolinium enhancement (C), and abnormal high T2 mapping value of left ventricular myocardium on 1.5 Tesla MRI (56.5 ± 7.18 milliseconds) (D: black arrows). (LV: Left ventricle).

Despite being clinically stable, the follow-up cardiac MRI images of the patient revealed persistent positive findings, which were suggestive of ongoing myocarditis. Below are the images illustrating the myocardial inflammation observed (Figure 1, 2).

## Discussion

In this study, we aimed at exploring the clinical course and cardiac MRI changes over a three-year follow-up period in patients diagnosed with SSc and myocarditis. We observed that 60% of patients had been female, and 70% had exhibited diffuse cutaneous skin involvement, consistent with typical patterns seen in SSc with myocarditis reported in the literature.^[3,10,11]^ All of our patients had been anti-topoisomerase I positive with 7 having diffuse skin involvement and 3 having limited skin involvement. One patient had both anti-topoisomerase I and anti-centromere antibodies. The presence of anti-topoisomerase I antibodies in SSc has been associated with a potential role in the continuous process of tissue healing and the development of unremitting fibrosis.^[12]^ As a result, patients, who had been anti-topoisomerase I positive, had shown more severe internal organ fibrosis.^[11]^ Additionally, the average age of our patient cohort aligned with the age range that had been commonly observed in SSc patients in previous studies.^[13]^ The mean duration of SSc at the diagnosis of myocarditis had been 4.8 ± 1.1 years after the onset of non-Raynaud symptoms of SSc.

In this study, initially, 7 patients had exhibited elevated levels of hs-cTnT. Following treatment with prednisolone alongside either cyclophosphamide or mycophenolate mofetil, normalization of cardiac enzyme levels was observed in 3 out of the initial 7 patients, and 4 patients showed improvement in their cardiac enzyme levels, although their levels did not return to normal. Three patients showed MRI scans consistent with myocarditis, displaying myocardial edema, hyperemia, and scars, despite having normal baseline levels of cardiac enzymes. Consistent with findings in the literature, Janardhanan *et al*. reported that only one-third of patients had demonstrated elevated cardiac enzymes. Consequently, when myocarditis is suspected clinically, it is advisable to pursue a cardiac MRI evaluation, incorporating T2-weighted, early gadolinium-enhanced, and late gadolinium-enhanced images, since this approach is considered to be the recommended method for diagnosis.^[14]^

When considering the cardiac MRI findings at diagnosis, all patients exhibited evidence of myocardial edema and hyperemia, indicating active inflammation within the myocardium. Additionally, some patients already had myocardial scars suggesting the chronicity of the disease before diagnosis. After treatment, all patients in our cohort maintained a stable NYHA Functional Class I or II throughout the follow-up period, with some even showing improvement to Class I. These findings suggested a trend toward clinical improvement over time in the majority of patients. However, despite this clinical improvement, our MRI assessments revealed that myocardial edema and hyperemia had persisted in all patients. After a three-year follow-up period, the cardiac MRI revealed stable myocarditis in four patients, improving myocarditis in four patients, and worsening myocarditis in two patients. Interestingly, there had been no complete resolution of myocarditis detected from follow-up cardiac MRI, although six patients had received steroids and immunosuppressant during follow-up. There were also reports suggesting a regression of signs of myocarditis from cardiac MRI in some patients with SSc who had been treated with steroids and/or immunosuppressants.^[2,15]^ Moreover, there was also a report of the normalization of acute myocarditis from cardiac MRI indices after treatment with prednisolone and azathioprine. ^[16]^ However, while these treatments have shown significant improvements, a complete and sustained resolution of myocarditis in SSc with these therapies has not been conclusively documented in the literature. A study of subclinical myocarditis in patients with SSc demonstrated an interval progression or even new occurrence of LGE without evidence of the complete resolution of myocarditis from serial cardiac MRI.^[17]^ Our observation along with other reports may underscore the chronic and persistent nature of myocarditis in SSc patients, highlighting the importance of ongoing monitoring and management strategies to address myocardial inflammation and its potential long-term implications.

Although many studies had suggested the benefits of treatment with steroids and/or immunosuppressants in the improvement of clinical status, cardiac biomarkers, and cardiac MRI findings in patients with SSc with myocarditis,^[15,16,18]^ such improvements were seemingly incomplete. In our study, the administration of steroids and immunosuppressants, which were actually given for ILD treatment, showed an inapparent trend in the improvement of myocarditis, in which 50% and 100% of patients with stable/improving and worsening myocarditis received such therapy, respectively. However, one could argue that not all patients in our study had received therapy, and though it was given in six out of ten patients, such therapy was not started immediately after the diagnosis had been made. Hence, a response to steroids and immunosuppressants in patients with SSc and myocarditis in our study could not be clearly drawn.

In terms of major cardiac events, interestingly, we did not find that any of the events had occurred during the follow-up. This is in contrast with the previous understanding that myocarditis in SSc should bear a poor prognosis.^[10,19]^ However, this could not be the case, since all of our patients were considered to have mild myocarditis, most of whom had been in NYHA FC II, had exhibited normal or mildly elevated hs-cTnT levels, and all had shown preserved LVEF and normal NT-proBNP levels at baseline. Therefore, not having cardiac events in an early stage or mild disease severity was reasonable, since such events usually occurred in patients with overt heart failure symptoms, high levels of cardiac biomarkers, and low LVEF.^[2]^ However, the follow-up period in this study might not have been long enough, and a longer follow-up of this particular group of patients might have added additional information about the prognosis of patients with SSc and mild myocarditis.

Association between changes in non-MRI parameters (NYHA FC and hs-cTnT levels) from the baseline to the last followup and the status of myocarditis were roughly sought in our study. However, it seems there had been no specific trend of association between the changes in non-MRI parameters and the status of myocarditis. Moreover, this observation could suggest against the use of stable non-MRI parameters for the predicting of disease outcomes or responses of treatment in patients with SSc and mild myocarditis.

Our study contains some limitations. Firstly, the sample size was small, and this prevented firm conclusions from being drawn. The enrollment included only patients with mild myocarditis, and enrolled patients with more severe phenotypes could probably yield other results. Treatment with corticosteroids and immunosuppressants was primarily aimed at the treatment of ILD, but not at treatment for myocarditis. This treatment was not given to all patients, all patients were not given the same regimen, or the treatment was started late after the initial cardiac MRI. It is conceivable that the administration of standardized treatment regimens for myocarditis early in the disease course across our patient cohort may have yielded different results. Finally, endomyocardial biopsy (EMB) was not performed to confirm the diagnosis of myocarditis and to compare it with the cardiac MRI findings. However, the role of EMB for the evaluation of myocarditis in SSc is limited,^[18]^ and it is truly inappropriate to perform EMB in patients, who are mildly symptomatic and who have no high-risk features of myocarditis.^[20]^

In summary, this was an exploratory prospective study using cardiac MRI to explore the clinical courses and outcomes of myocarditis in patients with SSc. Most of the patients had been mildly symptomatic, and all of the patients had been considered to have mild myocarditis. Throughout the study period, most patients had stable or improving myocarditis, whereas two patients developed worsening myocarditis according to cardiac MRI indices. However, there had seemingly not been any significant changes in functional class or in cardiac biomarkers at the end of follow-up, and importantly, no cardiac events had occurred. Further study with a larger sample size, with the probability of an earlier initiation of steroids and/or immunosuppressants, and with a longer period of follow-up is needed to elucidate the courses and outcomes of myocarditis in SSc, especially in the initially non-severe myocarditis phenotype.

## Conclusions

This cohort contained only patients with SSc, who considerably had mild myocarditis. We found that myocarditis in patients with SSc was chronic or persistent. Moreover, its outcomes on cardiac MRI could be stable, improving, or even worsening. However, there were seemingly neither significant nor predictable changes in clinical status or in the cardiac biomarker levels among each group of myocarditis status. Moreover, there were no major cardiac events that had occurred during the study. In this study, treatment with steroids and immunosuppressants had had no predictable effect on the progression of myocarditis in patients with SSc.
